# Ovulation triggers in unassisted reproduction, an old question still unanswered: a systematic review and meta-analysis

**DOI:** 10.5935/1518-0557.20250055

**Published:** 2025

**Authors:** Cristian Daniel Piccini, Julia Andressa Tessari, Murilo Gandon Brandão, Lucas Augusto Hauschild, Edison Capp, Helena von Eye Corleta

**Affiliations:** 1 Faculty of Medicine, Universidade Federal do Rio Grande do Sul, Porto Alegre, Rio Grande do Sul, Brazil; 2 Postgraduate Program in Gynecology and Obstetrics, Faculty of Medicine, Universidade Federal do Rio Grande do Sul, Porto Alegre, Brazil; 3 Department of Gynecology and Obstetrics, Hospital de Clínicas de Porto Alegre, Porto Alegre, Rio Grande do Sul, Brazil

**Keywords:** trigger, intrauterine insemination, ovulation, hCG, pregnancy

## Abstract

**Objective:**

To evaluate whether ovulation triggers improve pregnancy and/or ovulation rates in women with unexplained or anovulatory infertility undergoing ovulation induction and natural intercourse or intrauterine insemination (IUI), without increasing complications.

**Methods:**

We searched nine databases and grey literature from inception to August 29, 2023. Parallel-group randomized controlled trials (RCTs) including women with unexplained or anovulatory infertility undergoing ovulation induction and natural intercourse or IUI, comparing ovulation trigger versus placebo or no treatment, were included. Outcomes included pregnancy, ovulation, and complications. Risk of bias was assessed using the RoB 2 tool, and GRADE was applied for certainty of evidence. The Hartung-Knapp random-effects model was used to calculate odds ratio (ORs) (α=0.05). R packages “metafor”, “meta”, and “metasens” were used.

**Results:**

Six studies (1,218 women) were included; all studies used hCG as the trigger. For clinical pregnancy (n=855, 4 studies), OR=1.23 (95% CI: 0.91-1.66); for live birth (n=45, 2 studies), OR=0.92 (95% CI: 0.14-6.02); for miscarriage (n=89, 3 studies), OR=0.68 (95% CI: 0.20-2.35); for ovulation (n=311, 2 studies), OR=1.70 (95% CI: 0.84-3.43); and for multiple pregnancy (n=523, 2 studies), OR=2.56 (95% CI: 0.43-15.11). No subgroup analysis altered certainty of evidence and risk of bias was low.

**Conclusions:**

Current evidence is insufficient to recommend or refute ovulation triggers. hCG may increase ovulation, but effects on pregnancy outcomes remain uncertain.

## INTRODUCTION

Ovulation triggers (OT) are one of the cornerstones of modern assisted reproductive technology (ART). They work by pharmacologically mimicking the endogenous peak of gonadotropins that precedes the final maturation of the oocyte and its consequent release (Dosouto *et al.*, 2019). This strategy is not without risks, with ovarian hyperstimulation syndrome (OHSS) being the main adverse effect of using human chorionic gonadotropin (hCG) trigger, the first and most widely used OT. It is estimated that OHSS occurs in 0.3% to 6% of all stimulated cycles (Kumar *et al.*, 2011; Kupka *et al.*, 2014), with a mortality of approximately 1 in every 50,000 individuals (Diaz Ayllon *et al.*, 2023). Typically, OTs are used simultaneously with ovulation-inducing drugs, such as clomiphene citrate (CC) and human menopausal gonadotropin (hMG).

On the other hand, in the context of unassisted reproduction, which includes natural intercourse and, according to the definition of the American Center for Disease Control (CDC), intrauterine insemination (IUI) (Jain & Singh, 2025), the use of triggers is guided by uncertainty. Despite being widely used in practice as an additional way to increase pregnancy success rates, it is not clearly known whether their use is beneficial to patients, with several studies of limited methodological quality, including observational studies and crossover trials pointing to conflicting results. Furthermore, some clinical trials with small sample size claim that OTs do not work (Yilmaz *et al.*, 2006; George *et al.*, 2007), whilst others have reported the opposite (Harrison & O’Moore, 1983). In addition, although it is still unclear whether OTs are beneficial in this context, some studies have compared different OTs with each other, without including a placebo or no-intervention group (Sayyah-Melli *et al.*, 2012; Masrour & Azad, 2018). Finally, some systematic reviews (Kosmas *et al.*, 2007; Cantineau *et al.*, 2014; George *et al.*, 2014; Potapragada *et al.*, 2023) have highlighted the lack of evidence regarding the clinical benefits of OTs in certain non-ART contexts, emphasizing the need for additional high-quality studies. It is important to highlight that Potapragada *et al.* (2023)conducted a meta-analysis that included both randomized controlled trials and observational studies, focusing on IUI cycles regardless of infertility etiology. More than a decade has passed since some of these recommendations were made.

Therefore, it is still not clear whether OT strategy is beneficial for patients trying to conceive by methods other than ART. Here, we aim to evaluate whether OTs improve pregnancy and/or ovulation rates in women with unexplained or anovulatory infertility undergoing ovulation induction by natural intercourse or IUI, without increasing complications.

## MATERIAL AND METHODS

This systematic review follows the guidelines of the Cochrane Handbook for Systematic Reviews of Interventions (Higgins *et al.*, 2023) (hereinafter referred to simply as the “Cochrane Handbook”), the Preferred Reporting Items for Systematic reviews and Meta-Analyses (PRISMA) 2020 statement (Page *et al.*, 2021), and is registered on PROSPERO under ID 257205 (Piccini *et al.*, 2021). Since the data reviewed are public, this study did not require Institutional Review Board (IRB) approval.

### Search Strategy and Study Selection

Literature searches were conducted in PubMed, Embase, CENTRAL, Scopus, Web of Science, LILACS, ClinicalTrials.gov, OpenGrey and Google Scholar (last updated, August 29, 2023; except for CENTRAL (last update, April 1, 2021), which was no longer available to the reviewers, and OpenGrey (last update, May 12, 2021), which was discontinued). Moreover, in search of additional studies, citation index databases (Scopus and Web of Science) were used, and the reference lists of included articles and relevant systematic reviews were checked. Full search strategies are available in Supplementary Appendix 1. Three reviewers applied the inclusion and exclusion criteria. Two reviewers (JAT and MGB) independently screened records for inclusion, blinded to each other’s decisions, and a third reviewer (CDP) checked the decisions. Any disagreements were resolved by discussion until consensus was reached. Kappa statistics was used to measure (prior to checking and discussion) agreement for study selection. A kappa value of 0.75 was obtained, which translates into substantial interrater agreement. Rayyan software was used for the selection and recording of decisions.

### Inclusion and Exclusion Criteria

Parallel group randomized controlled trials (RCTs) reporting on women with anovulatory or unexplained infertility undergoing ovulation induction followed by natural intercourse or IUI under an OT regimen *versus* placebo or no treatment, that measured at least one of our outcomes of interest. There were no restrictions regarding language, publication status, country, race, ethnicity, age or publication date. Only studies with participants undergoing natural intercourse or intrauterine insemination were included. The listed outcomes constitute outcomes of interest within the included studies and not decision criteria for their respective inclusion. However, studies that did not measure any outcomes of interest, as well as studies without full-text availability, were excluded. Studies with a crossover design and studies in which the ovulation trigger was administered in all intervention arms were also excluded. Additional details regarding inclusion and exclusion criteria may be found in the study protocol (Piccini *et al.*, 2021).

### Data Extraction

Four researchers participated in the data extraction process. For each study, two researchers (among JAT, MGB, and LAH) independently extracted data using Systematic Review Data Repository-Plus (SRDR+), and a third reviewer (CDP) checked and consolidated the extracted data. A kappa value of 0.84 was obtained, indicating almost perfect interrater agreement. In case of disagreements, the three reviewers discussed until consensus was reached. Original authors were contacted whenever there was missing data. The following data were extracted for each study: study methods (ie., full description of random sequence generation, allocation sequence concealment, blinding and likelihood of reporting bias and other biases), participants (ie. setting, region, eligibility criteria and baseline characteristics, such as age, ethnicity, diagnostic criteria, comorbidity and socio-economic status), intervention (ie., components, routes of delivery, doses, timing, frequency, intervention protocols, length of intervention, co-interventions and definition of “control” groups), outcomes (ie., names of all outcomes measured and, for pre-specified outcomes, evidence that the outcome was assessed, measurement tool or instrument, specific metric, method of aggregation and timing of outcome measurements), results (ie., for each group and subgroup (pre-specified in the protocol (Piccini *et al.*, 2021), number of participants randomly assigned and included in the analysis, number of participants who withdrew, were lost to follow-up or were excluded, summary data, and between-group estimates quantifying the effect of the intervention on the outcome (as well as their precision), key conclusions and reference to other relevant studies.

### Outcomes

The main outcomes were clinical pregnancy, live birth, and miscarriage rates. Additional outcomes were ovulation, multiple pregnancy, preterm delivery, ovarian hyperstimulation syndrome, multiple birth, perinatal death/mortality, adverse events, and mortality. Clinical pregnancy was evaluated by ultrasonographic visualization of one or more gestational sacs or by definitive clinical signs of pregnancy. Live birth was defined as the complete expulsion or extraction from a woman of a product of fertilization, after 22 completed weeks of gestational age, which, after separation, breathes or shows any other evidence of life, such as heart beat, umbilical cord pulsation or definite movement of voluntary muscles, irrespective of whether the umbilical cord has been cut or the placenta is attached. Miscarriage was defined as the spontaneous loss of an intra-uterine pregnancy prior to 22 completed weeks of gestational age. Ovulation was evaluated by serum progesterone level or presumed by serial transvaginal ultrasound. Multiple pregnancy was defined as a pregnancy with more than one embryo or fetus, diagnosed by ultrasonographic visualization of more than one gestational sac. As defined in the protocol, the “Summary of findings” (SoF) table includes the main outcomes and the first four additional outcomes. Multiplicity of outcomes was handled using a “decision rules” approach, blinded to the results. The following hierarchy was used: (1) the study’s primary outcome; (2) the outcome that closely resembles the definition provided here for our outcomes; (3) random selection.

### Quality Assessment

For each outcome of each study included in this review, risk of bias assessment was performed independently by two assessors (among JAT, MGB and LAH), using version 2 of the Cochrane risk-of-bias tool for randomized trials (RoB 2). A third reviewer (CDP) checked the decisions and consolidated data. Any disagreements were resolved by discussion.

The certainty of the body of evidence was assessed using the Grading of Recommendations, Assessment, Development, and Evaluation (GRADE) approach. A minimally important difference (MID) (Zeng *et al.*, 2022) of 5% was used for ovulation, clinical pregnancy, and multiple pregnancy. The Summary of Findings (SoF) table was created through (GRADEpro GDT software, 2004), available from gradepro.org.

### Strategy for Data Analysis

Our primary statistical analyses consisted of combining, for each outcome, the odds ratios (ORs) for individual studies using the modified Hartung-Knapp method for random-effects (RE) model. The study protocol predefined situations in which a fixed-effect (FE) model would be used instead, but these did not happen. An alpha level of 0.05 was used. Contour-enhanced funnel plots were generated for the primary analyses. Assessment of risk of bias in syntheses owing to missing results were performed for the outcomes presented in the SoF table, following the framework provided in Cochrane Handbook (Page *et al.*, 2023). Subgroup analyses were conducted based on the type of infertility (ie. anovulatory vs unexplained). Other subgroup analyses were pre-specified in the protocol, but the data reported by the included studies did not allow us to conduct them. Studies at high risk of bias were not included in our analyses; sensitivity analyses were conducted to assess whether that had an impact on our results. We used “metafor” and “meta” R packages (along with the complement “metasens”). The main analyses were conducted with the “metafor” package, while “metasens” was used to perform the sensitivity analyses described later.

Heterogeneity was assessed using χ^2^ test. A *p*-value of less or equal to 0.10 determined statistical significance. Between-study variance (τ^2^) was estimated using Paule-Mendel approach. Inconsistency was quantified with I^2^ statistic. We pre-specified that a random-effects (RE) meta-analysis would not be conducted if I^2^ was greater than 75%. Nonetheless, other aspects, such as the magnitude of effect and the *p*-value for Cochran’s Q, would also be considered in this decision.

Higgins et al.’s (Higgins *et al.*, 2008) Informative Carrying-Forward Analysis for randomized trials (ICA-r) approach for imputation of missing data was used. For this purpose, as defined in the study protocol, the ICA-0 approach was applied if missingness was due to lack of benefit; ICA-pC if it was due to adverse events, refusal to follow the protocol, withdrawal of consent, change of treatment or desire for (or indication of) in vitro fertilization; and ICA-p if it was due to loss to follow-up, move, partner’s disease, other health impairment independent of the study or another reason. We used weighting scheme W4 to derive standard errors. Moreover, sensitivity analyses were conducted according to the authors’ recommended strategy. For these, a selection of combinations of informative missingness odds ratio (IMOR) for the experimental group (IMOR_E_) and control group (IMOR_C_) were used, calculated based on the vision of experts in the field for the missing data in question. The Gamble and Hollis’s (Gamble & Hollis, 2005) approach was also used in these analyses. Finally, the weighting for these sensitivity analyses was calculated in R using the “metasens” package.

## RESULTS

**Figure 1:**
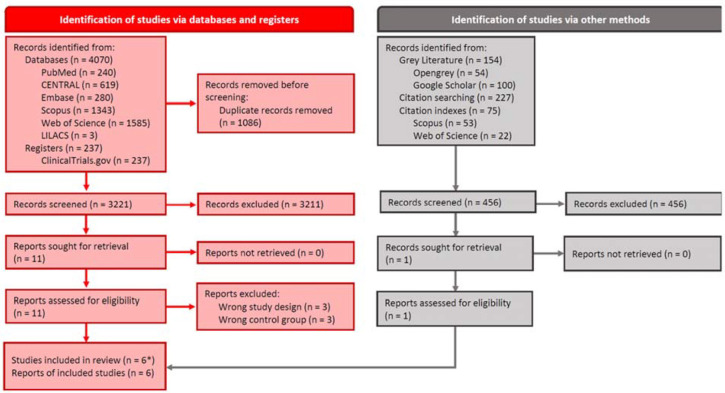
PRISMA (Preferred Reporting Items for Systematic Reviews and Meta-Analyses) flow diagram of the article selection process. * There is no evidence that supports that one of these studies was conducted beyond the recruitment phase.

**Figure 2:**
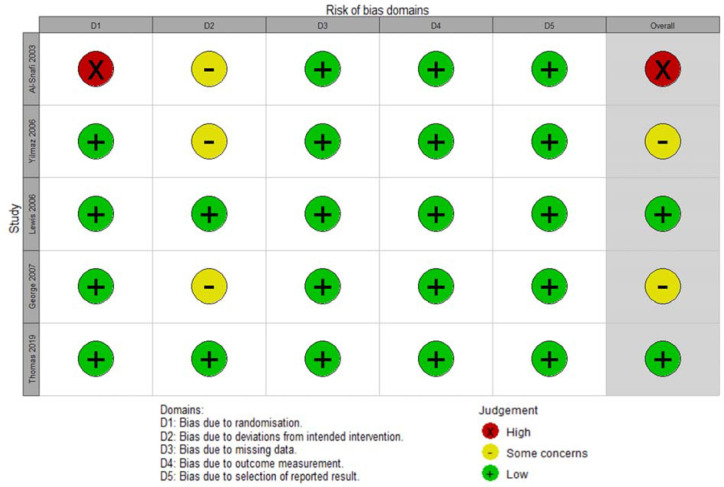
Risk of bias assessment according to the Cochrane Risk of Bias tool for randomized trials (RoB 2). For each study, all outcomes of interest received the same judgments and are therefore presented only once.

Initial searches retrieved 4,763 records. After removing duplicates and applying inclusion and exclusion criteria, six studies (Al-Snafi *et al.*, 2003; Lewis *et al.*, 2006; Yilmaz *et al.*, 2006; George *et al.*, 2007; El-khayat, 2017; Thomas *et al.*, 2019) were included in our review. Of these, one is an ongoing trial (El-khayat, 2017). Figure 1 presents the PRISMA flow diagram of the article selection process. Of the remainder, one was considered to have “high risk of bias”, two “some concerns” and two “low risk of bias” (Figure 2). A “Characteristics of excluded studies” table can be found in Supplementary Appendix 2. This table provides details regarding the specific reasons for the exclusion of studies (Harrison & O’Moore, 1983; Zreik *et al.*, 1999; Sayyah-Melli *et al.*, 2012; Masrour & Azad, 2018; Thabet *et al.*, 2024) that the reader could expect to see among the included studies. Supplementary Appendix 5 shows key aspects of risk of bias judgements for the included studies.

### Study characteristics

A total of 1,218 participants were included in our analyses. When considering only studies with a sufficiently low risk of bias to be included in our primary analyses, the largest number of participants obtained was 855, corresponding to the number of participants for the “clinical pregnancy” outcome. Of the included studies, two were conducted in India, one in Iraq, one in Turkey, and one in the United States. Clomiphene citrate (CC) was the most commonly used ovulation induction agent, but human menopausal gonadotropin (hMG) was used in one study, and a combination of bromocriptine + CC in another one. The trigger used was hCG in all studies, in doses of 5,000 or 10,000 IU, administered after a follicle of at least 17-21mm was visualized on ultrasound or on the day of ovulation. For all studies, the control group was “no intervention” (ie., no placebo was used). Individually, the included studies failed to demonstrate benefits or harms compared to “no intervention” (ie., no trigger). Supplementary Appendix 3 displays the main characteristics of included studies.

### Meta-analysis

**Figure 3:**
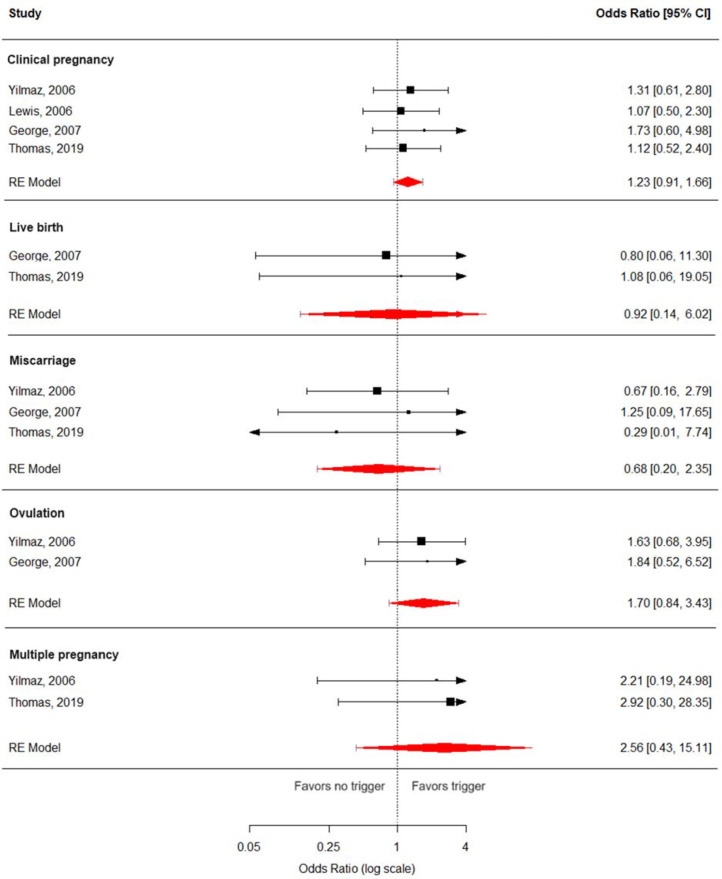
Forest plots for the primary analyses. For all outcomes, *I*^2^ = 0, and *p*-values for Cochran’s Q were within the range of statistical non-significance.

For all the below-mentioned analyses (Figure 3), an OR higher than 1 indicates that, when comparing trigger *versus* no trigger, the condition or event is more likely to occur in the trigger (intervention) group. Results for sensitivity analyses with missing data, following Higgins et al.’s (Higgins *et al.*, 2008) recommended strategy, are presented in Table 1.

**Table 1:** Sensitivity analyses following Higgins et al. (2008). IMORE and IMORC were calculated based on expert opinion regarding the missing data from the included studies. Effect estimates were calculated in “R” using the “metasens” package. There were no missing data for the outcomes “Miscarriage” and “Live birth”. OR: odds ratio, ICA: imputed case analysis, IMOR: informative missingness odds ratio for experimental group (E) or control group (C).

		Yilmaz *et al.*, 2006	Lewis *et al.*, 2006	George *et al.*, 2007	Thomas *et al.*, 2019	Meta-analysis	
		OR	OR	OR	OR	Pooled OR (95% CI)	I^2^
Clinical pregnancy							
Reference analysis	ICA-r	1.31	1.07	1.73	1.12	1.23 (0.91, 1.66)	0%
Sensitivity analysis	IMOR_E_ = 2, IMOR_C_ = 2	1.38	0.93	1.71	1.18	1.22 (0.83, 1.77)	0%
	IMOR_E_ = 0.5, IMOR_C_ = 0.5	1.25	1.22	1.74	1.11	1.26 (0.97, 1.63)	0%
	IMOR_E_ = 0.5, IMOR_C_ = 2	1.21	0.81	1.68	0.91	1.04 (0.65, 1.66)	0%
	IMOR_E_ = 2, IMOR_C_ = 0.5	1.42	1.41	1.77	1.44	1.47 (1.27, 1.69)	0%
	Gamble-Hollis	1.31	1.08	1.73	1.14	1.35 (0.98, 1.86)	0%
Ovulation							
Reference analysis	ICA-r	1.63		1.84		1.70 (0.84, 3.43)	0%
Sensitivity analysis	IMOR_E_ = ∞, IMOR_C_ = ∞	1.79		1.81		1.80 (1.67, 1.94)	0%
	IMOR_E_ = 0.5, IMOR_C_ = 0.5	1.53		1.85		1.63 (0.52, 5.09)	0%
	IMOR_E_ = 0.5, IMOR_C_ = ∞	1.46		1.73		1.54 (0.55, 4.30)	0%
	IMOR_E_ = ∞, IMOR_C_ = 0.5	1.88		1.94		1.90 (1.57, 2.30)	0%
	Gamble-Hollis	1.63		1.84		1.70 (0.83, 3.52)	0%
Multiple pregnancy							
Reference analysis	ICA-r	2.21			2.92	2.56 (0.43, 15.11)	0%
Sensitivity analysis	IMOR_E_ = 2, IMOR_C_ = 2	2.36			3.30	2.82 (0.34, 23.38)	0%
	IMOR_E_ = 0, IMOR_C_ = 0	2.03			3.03	2.51 (0.20, 31.82)	0%
	IMOR_E_ = 0, IMOR_C_ = 2	1.94			2.27	2.11 (0.78, 5.74)	0%
	IMOR_E_ = 2, IMOR_C_ = 0	2.47			4.40	3.36 (0.09, 129.73)	0%
	Gamble-Hollis	2.21			3.19	2.48 (0.28, 22.00)	0%

### Primary outcomes

Collectively, studies yielded a pooled OR of 1.23 (95% CI: 0.91, 1.66; *p*=0.1122; *I*^2^: 0%) for clinical pregnancy. Sensitivity analyses including the study with high risk of bias [24] resulted in an OR of 1.16 (95% CI: 0.97, 1.40; *p*=0.0876; I^2^: 0%). Subgroup analyses were conducted for the type of infertility, yielding an OR of 1.44 (95% CI: 0.26, 7.85; *p*=0.2244; *I*^2^: 0%) for clinical pregnancy in patients with anovulatory infertility (1.20 [95% CI: 0.75, 1.91; *p*=0.2408; I^2^: 0%], including the study with high risk of bias), and 1.09 (95% CI: 0.81, 1.48, *p*=0.1631, I^2^: 0%) in the scenario where a majority of infertility unexplained. The first scenario also used natural intercourse as the exclusive reproductive method, while in the latter scenario, IUI was used exclusively.

Regarding the other primary outcomes, an OR of 0.92 (95% CI: 0.14, 6.02; *p*=0.6632; I^2^: 0%) was obtained for live birth when comparing trigger versus no trigger, and an OR of 0.68 (95% CI: 0.20, 2.35; *p*=0.3107; I^2^: 0%) for miscarriage. Subgroup analyses including only anovulatory infertility resulted in an OR of 0.77 (95% CI: 0.03, 21.76; *p*=0.5000; I^2^: 0%) for miscarriage. Similar to the clinical pregnancy outcome, this scenario also used, coincidentally, only natural intercourse as the reproductive method. Only one study that reported this outcome recruited patients with unexplained infertility, hence no pooled OR is presented for this setting. All analyses regarding primary outcomes yielded p values for Cochran’s Q in the range of statistical non-significance.

### Secondary outcomes

Collectively, studies yielded a pooled OR of 1.70 (95% CI: 0.84, 3.43; *p*=0.0665; *I*^2^: 0%) for ovulation, and 2.56 (95% CI: 0.43, 15.11; p=0.0938; I^2^: 0%) for multiple pregnancy. The included studies did not measure the other secondary outcomes foreseen in our protocol. Similarly to what was observed for the primary outcomes, all analyses regarding secondary outcomes yielded P values for Cochran’s Q in the range of statistical non-significance.

## DISCUSSION

**Figure 4:**
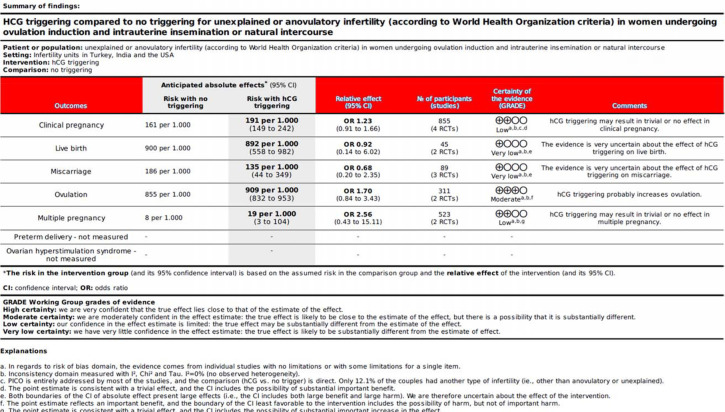
Summary of findings (SoF).

In this systematic review, we aimed to evaluate whether the use of OTs in patients with anovulatory or unexplained infertility is beneficial in the context of natural intercourse or IUI. As demonstrated in detail in the SoF table (Figure 4), when the GRADE approach is used to interpret the results, it is clear that the use of the trigger probably leads to an increased ovulation, but for now the results do not allow one to conclude whether this translates into clinical benefit for patients. It is worth mentioning that the lack of evidence of effect is not evidence of lack of effect. Following the Cochrane Handbook, we avoid using the terms “statistically significant” or “statistically non-significant”. These labels are based on arbitrary thresholds and may not reflect the clinical or policy significance of the findings (Schünemann *et al.*, 2023).

The certainty of evidence for subgroup analyses is the same as for primary analyses, except for clinical pregnancy in anovulatory infertility, which is further downgraded to “very low”. Identically to the primary analyses, subgroup analyses for the type of infertility or reproductive method used, when possible, do not allow us to confirm whether the use of the trigger brings clinical benefits. Although subgroup analyses offered some exploratory insights regarding the type of infertility and the reproductive method used, the current evidence remains insufficient to establish any clinically meaningful differences. These findings should be interpreted cautiously, as they mirror the uncertainties observed in the primary analyses. Future studies specifically designed to address these subgroup variations are necessary to clarify their potential clinical implications.

In our analyses, we used the ICA-r approach of Higgins *et al.* (2008) for missing data, in which imputation is made according to the available reasons for missingness, determining appropriate IMORs. This allows for different imputation strategies to be used, enabling “best-guess” primary analyses to be transparently obtained. As shown in Table 1, when IMORs are varied over plausible ranges, the results can be sufficiently different to change the certainty of the evidence for all outcomes with missing data. On the other hand, when the inflated confidence intervals of Gamble & Hollis (2005) are used, the results do not change enough to alter the respective interpretation. This indicates that the conclusions of this review are robust enough for the effect of allowing for missing data on the standard errors (and hence weights) of the estimates, but not for the effect of allowing for missing data on the effect estimates from the individual studies.

Our findings regarding clinical pregnancy are consistent with those reported by Potapragada *et al.* (2023). However, our review differs methodologically in several important aspects. We included only parallel-group randomized controlled trials and focused specifically on women with anovulatory or unexplained infertility undergoing ovulation induction followed by natural intercourse or IUI. Crossover trials and studies lacking appropriate control groups were excluded. Furthermore, we applied advanced statistical methods for handling missing data (ICA-r) and evaluated the certainty of evidence using the GRADE approach, following the latest Cochrane Handbook guidelines.

Other previous reviews, although addressing similar questions, differ substantially in design and scope Cantineau *et al.* (2014) limited the study to RCTs but included crossover trials, also in the context of IUI. Kosmas *et al.* (2007) also focused on IUI and allowed study designs other than RCTs, concluding that the use of OTs did not show a clinically important effect when compared to spontaneous ovulation.

The meta-analysis conducted by George *et al.* (2014) aimed to assess the effectiveness of ovulation triggers in women with anovulatory infertility undergoing timed intercourse. The authors included randomized controlled trials and concluded that there was insufficient certainty of evidence to support or refute the routine use of urinary hCG in this population. Despite this cautious interpretation, the review stated that “giving women on clomiphene citrate additional urinary hCG may not increase their chances of ovulating,” which contrasts with the findings of our meta-analysis. This divergence appears to be the result of a methodological error identified in the prior review. Specifically, during the data extraction and analysis of the study by Yilmaz *et al.* (2006), the authors of the George *et al.* (2014) meta-analysis mistakenly reversed the group assignments: the results from the intervention group were entered as those from the control group, and vice versa. This misclassification affected the direction of the effect estimates and led to misleading conclusions. Moreover, this inversion was not limited to ovulation outcomes and may have impacted the interpretation of other key endpoints as well. By addressing such discrepancies and applying rigorous methods to data extraction and synthesis, our review aims to provide a more accurate assessment of the current evidence and highlight the need for further high-quality trials to clarify the role of ovulation triggers in unassisted reproduction.

### Risk of bias due to missing results in syntheses

One of the first aspects to be considered when analyzing the risk of bias in summary effect estimates due to missing results is the unreported results in the identified studies (also known as “known unknowns”). In this sense, it is immediately identified that one of the studies included in our review does not have available results (El-khayat, 2017). It was first posted on clinicaltrials.gov in October, 2014, and last updated in February, 2017. Actually, there is no evidence that supports that this study was conducted beyond the recruitment phase. The reviewers tried to contact the principal investigator of this trial, but no response was obtained. Since this is a study that was sponsored by a public university, we are less inclined to assume that conflicts of interest were the reason for the possible discontinuation of the trial. Equivalently, it is not reasonable to suspect that results were missing in the other included trials because of the *p*-value, magnitude or direction of the result (Page *et al.*, 2023).

A second aspect to be considered is the missing results from additional studies (also known as “unknown unknowns”). In this sense, what stands out, first of all, is the comprehensiveness of the searches carried out, which went far beyond searching one or two standard databases, and was not restricted to any specific language. Another important aspect when discussing “unknown unknowns”, however, is beyond the control of reviewers: publication bias. To address this aspect, contour-enhanced funnel plots (Peters *et al.*, 2008) were generated for the primary analyses (Supplementary appendix 4). As can be seen, for the outcomes “Clinical pregnancy”, “Ovulation” and “Multiple pregnancy”, there is a suggestion of missing studies on the left-hand side of the plot, where results would be unfavorable to the experimental intervention (at least for the first two outcomes) and broadly in the area of non-significance, which conceptually increases the likelihood that the asymmetry is due to publication bias. However, even in this case, other explanations for the asymmetry, such as artifacts and chance alone, should still be considered (Sterne *et al.*, 2011; Page *et al.*, 2023). In fact, when we take into account that all published studies are in the zone of lack of statistical significance, that their authors do not conclude in favor of the use of the trigger, and that conflicts of interest (both declared and evaluated by the RoB 2 tool) appear not be present in these studies, it seems to us to be incoherent to consider publication bias as the first hypothesis. Finally, due to the low number of studies (fewer than 10), tests for funnel plot asymmetry are not recommended. Nor is comparing FE and RE estimates appropriate, since there is no evidence of between-study heterogeneity (*I*^2^>0). Our overall judgment is therefore of “low risk of bias” due to missing results in syntheses.

### Differences between protocol and review

Initially, we intended to perform statistical analyses using only the R package “meta” and its complement “metasens”. Although the latter includes several of the methods described by Higgins *et al.* (2023), the “ICA-r” approach is not present. It was therefore necessary to conduct the analyses using the “metafor” package, in order to enable compliance with the imputation and weighting method foreseen in the study protocol (Piccini *et al.*, 2021).

Furthermore, during the selection of studies for inclusion, we came across two studies whose majority of participants, but not all, met our inclusion criteria. The first one (Thomas *et al.*, 2019) randomized 396 patients, of which 86.99% had anovulatory or unexplained infertility, thus meeting our inclusion criteria for the type of infertility. For the remainder of the participants, the cause of infertility was “male factor” or “endometriosis”, therefore not meeting our inclusion criteria. Similarly, a second study (Lewis *et al.*, 2006) randomized 150 patients, of which 64.67% had unexplained infertility, and the remainder “male factor”, “endometriosis”, “cervical factor” or “tubal/pelvic factor”. In the “Participants” section of the protocol (Piccini *et al.*, 2021), we defined that “Studies which include only a subset of relevant participants, and whose data cannot be retrieved, will not be part of the summary of effect estimates. Sensitivity analyses will be performed.” However, at that moment, we expected a larger number of studies/participants to be available. In practice, not including these two studies would mean reducing the number of participants included in our review from 855 to 309, that is, a reduction of 63.86%, excluding the study with a high risk of bias. Therefore, following the objectives of our review, and due to the fact that the majority of patients in these two studies met our inclusion criteria, we chose not to follow the protocol at this point, and include these studies in the primary analyses. As mentioned in Cochrane Handbook, “post-hoc decisions about inclusion or exclusion of studies should maintain faith with the objectives of the review rather than with arbitrary rules.”(McKenzie *et al.*, 2024). Sensitivity analyses excluding these studies are similar to subgroup analyses for anovulatory infertility. If these studies were not included, it would not be possible to present pooled ORs for the outcomes “multiple pregnancy” and “live birth”, and the certainty of the evidence for “clinical pregnancy” would be further downgraded to “very low”. In this case, the general conclusions of the systematic review would remain unchanged, with minor adjustments regarding these specific outcomes, in order to adapt to the new quality of evidence.

### Limitations

Our systematic review is limited by the low number of high-quality clinical trials on the topic. This, combined with the relatively small number of patients included in the studies, results in a low overall sample size for the review. As a result, the level of certainty of evidence of our results is limited. Moreover, not all outcomes specified in our protocol were measured. Finally, we were unsuccessful in obtaining results from the subset of participants with anovulatory or unexplained infertility in the studies by Thomas *et al.* (2019) and Lewis *et al.* (2006) despite attempts to contact the authors. Although we tend to believe that this limitation is unlikely to change the overall conclusions of the review, it constitutes an additional limitation.

### Strengths and implications for future research

This systematic review stands out for the breadth of searches conducted, which include six main databases, a clinical trials registry, gray literature, reference lists of included studies and other relevant systematic reviews, as well as citation index databases. Furthermore, it strictly follows the practices recommended by the Cochrane Handbook and the most recent updates of the GRADE approach, in addition to incorporating more advanced statistical methods for meta-analysis for the imputation of missing data than those provided by standard software, with the aim of obtaining results closer to what would be observed in reality. This level of care in defining methods increases the precision of summary effect estimates and reduces the risk of bias due to missing results in syntheses. Finally, it supports one a proper justification for the future execution of new high-quality RCTs on this topic.

## CONCLUSIONS

To date, the data available from trials already conducted are not sufficient to recommend or refute the use of OTs in women with anovulatory or unexplained infertility in the context of unassisted reproduction. Our findings indicate that hCG triggering probably increases ovulation, but may result in trivial or no effect on clinical pregnancy and multiple pregnancy, and that the evidence is very uncertain regarding the effect of hCG triggering on live birth and miscarriage. More parallel-group RCTs are needed to answer this long-standing question.

## Figures and Tables

**Supplementary Appendix 4:**
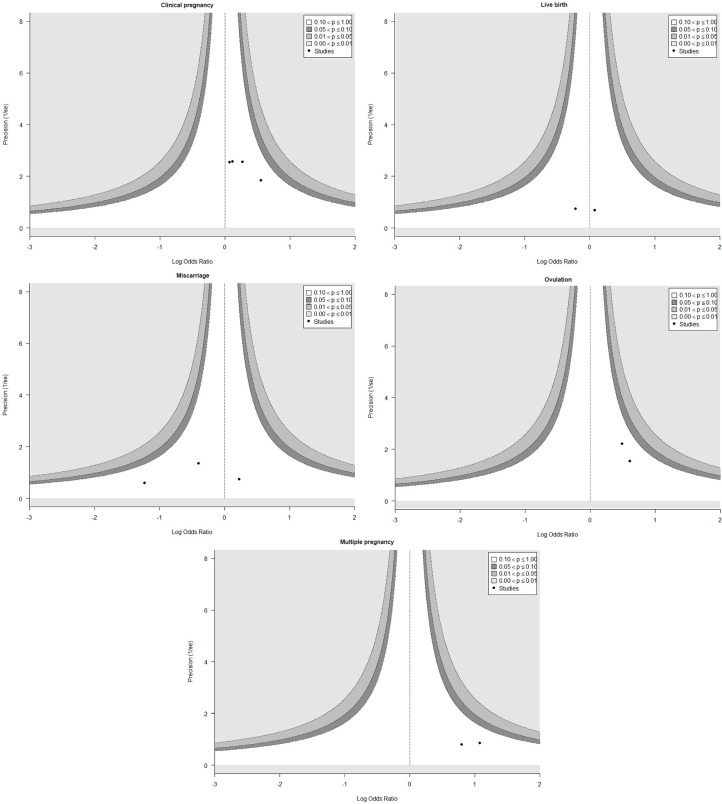
Contour-enhanced funnel plots the primary analyses. There is a suggestion of missing studies on the left-hand side of the plots of “Clinical pregnancy”, “Ovulation” and “Multiple pregnancy”.

## Supplementary Material

### Supplementary Appendix 1. Full database search strategies

#### PubMed

(Chorionic Gonadotropin[mh] OR Recombinant HCG[tw] OR Leuprolide[mh] OR A-43818[tw] OR Enantone[tw] OR Leuprolide*[tw] OR Leuprorelin[tw] OR Lupron[tw] OR TAP-144[tw] OR Buserelin[mh] OR Buserelin*[tw] OR HOE-766[tw] OR Suprecur[tw] OR Suprefact[tw] OR Tiloryth[tw] OR Nafarelin[mh] OR Nafarelin*[tw] OR Synarel[tw] OR Triptorelin Pamoate[mh] OR Triptorelin*[tw] OR AY-25650[tw] OR D-Trp-6-LH-RH[tw] OR Decapeptyl*[tw] OR Trelstar[tw] OR Gonadotropin-Releasing Hormone/agonists[mh] OR Luteinizing Hormone/AD[mh] OR Interstitial Cell-Stimulating Hormone[tw] OR Luteoziman[tw] OR Luteozyman[tw] OR Lutropin[tw] OR Kisspeptins[mh] OR Kisspeptin*[tw] OR KiSS-1 Metastasis Suppressor[tw] OR Metastasis Suppressor KiSS-1[tw] OR Metastin*[tw])

AND

(infertility, female[mh] OR female infertility[tw] OR anovulation[mh] OR anovulat*[tw])

AND

(randomized controlled trial[pt] OR controlled clinical trial[pt] OR randomized[tiab] OR placebo[tiab] OR drug therapy[sh] OR randomly[tiab] OR trial[tiab] OR groups[tiab]) NOT (animals [mh] NOT humans [mh])

We utilized Cochrane Highly Sensitive Search Strategy for identifying randomized trials in MEDLINE: sensitivity-maximizing version (2008 revision); PubMed format

**Filter:** “randomized clinical trial”

#### CENTRAL

ID Search Hits

#1 MeSH descriptor: [Chorionic Gonadotropin] explode all trees 906

#2 MeSH descriptor: [Leuprolide] explode all trees 698

#3 MeSH descriptor: [Buserelin] explode all trees 292

#4 MeSH descriptor: [Nafarelin] explode all trees 78

#5 MeSH descriptor: [Triptorelin Pamoate] explode all trees 460

#6 MeSH descriptor: [Gonadotropin-Releasing Hormone] explode all trees 2671

#7 MeSH descriptor: [Luteinizing Hormone] explode all trees 1633

#8 MeSH descriptor: [Kisspeptins] explode all trees 13

#9 “Recombinant HCG” OR “A-43818” OR Enantone OR Leuprolide* OR Leuprorelin OR Lupron OR “TAP-144” OR Buserelin* OR “HOE-766” OR Suprecur OR Suprefact OR Tiloryth OR Nafarelin* OR Synarel OR Triptorelin* OR “AY-25650” OR “D-Trp-6-LH-RH” OR Decapeptyl* OR Trelstar OR “Interstitial Cell-Stimulating Hormone” OR Luteoziman OR Luteozyman OR Lutropin OR Kisspeptin* OR “KiSS-1 Metastasis Suppressor” OR “Metastasis Suppressor KiSS-1” OR Metastin* 2946

#10 #1 OR #2 OR #3 OR #4 OR #5 OR #6 OR #7 OR #8 OR #9 5766

#11 MeSH descriptor: [Infertility, Female] explode all trees 1486

#12 MeSH descriptor: [Anovulation] explode all trees 150

#13 “female infertility” OR anovulat* 2421

#14 #11 OR #12 OR #13 3531

#15 #10 AND #14 641

**Filter:** “trials”

#### Embase

(“Chorionic Gonadotropin”/exp OR “Recombinant HCG”:ti,ab,kw OR leuprorelin/exp OR “A-43818”:ti,ab,kw OR Enantone:ti,ab,kw OR Leuprolide*:ti,ab,kw OR Leuprorelin:ti,ab,kw OR Lupron:ti,ab,kw OR “TAP-144”:ti,ab,kw OR Buserelin/exp OR Buserelin*:ti,ab,kw OR “HOE-766”:ti,ab,kw OR Suprecur:ti,ab,kw OR Suprefact:ti,ab,kw OR Tiloryth:ti,ab,kw OR Nafarelin/exp OR Nafarelin*:ti,ab,kw OR Synarel:ti,ab,kw OR triptorelin/exp OR Triptorelin*:ti,ab,kw OR “AY-25650”:ti,ab,kw OR “D-Trp-6-LH-RH”:ti,ab,kw OR Decapeptyl*:ti,ab,kw OR Trelstar:ti,ab,kw OR “gonadorelin agonist”/exp OR “Luteinizing Hormone”/exp OR “Interstitial Cell-Stimulating Hormone”:ti,ab,kw OR Luteoziman:ti,ab,kw OR Luteozyman:ti,ab,kw OR Lutropin:ti,ab,kw OR kisspeptin/exp OR Kisspeptin*:ti,ab,kw OR “KiSS-1 Metastasis Suppressor”:ti,ab,kw OR “Metastasis Suppressor KiSS-1”:ti,ab,kw OR Metastin*:ti,ab,kw)

AND

(“female infertility”/exp OR “female infertility”:ti,ab,kw OR anovulation/exp OR anovulat*:ti,ab,kw)

AND

(‘randomized controlled trial’/exp OR ‘controlled clinical trial’/exp OR random*:ti,ab OR ‘randomization’/exp OR ‘intermethod comparison’/exp OR placebo:ti,ab OR compare:ti OR compared:ti OR comparison:ti OR ((evaluated:ab OR evaluate:ab OR evaluating:ab OR assessed:ab OR assess:ab) AND (compare:ab OR compared:ab OR comparing:ab OR comparison:ab)) OR ‘open label’:ti,ab OR ((double:ti,ab OR single:ti,ab OR doubly:ti,ab OR singly:ti,ab) AND (blind:ti,ab OR blinded:ti,ab OR blindly:ti,ab)) OR ‘double blind procedure’/exp OR ‘parallel group*’:ti,ab OR crossover:ti,ab OR ‘cross over’:ti,ab OR ((assign*:ti,ab OR match:ti,ab OR matched:ti,ab OR allocation:ti,ab) AND (alternate:ti,ab OR group*:ti,ab OR intervention*:ti,ab OR patient*:ti,ab OR subject*:ti,ab OR participant*:ti,ab)) OR assigned:ti,ab OR allocated:ti,ab OR (controlled:ti,ab AND (study:ti,ab OR design:ti,ab OR trial:ti,ab)) OR volunteer:ti,ab OR volunteers:ti,ab OR ‘human experiment’/exp OR trial:ti) NOT (random*:ti,ab AND sampl*:ti,ab AND (‘cross section*’:ti,ab OR questionnaire*:ti,ab OR survey*:ti,ab OR database*:ti,ab) NOT (‘comparative study’/exp OR ‘controlled study’/exp OR ‘randomised controlled’:ti,ab OR ‘randomized controlled’:ti,ab OR ‘randomly assigned’:ti,ab) OR (‘cross-sectional study’/exp NOT (‘randomized controlled trial’/exp OR ‘controlled clinical study’/exp OR ‘controlled study’/exp OR ‘randomised controlled’:ti,ab OR ‘randomized controlled’:ti,ab OR ‘control group*’:ti,ab)) OR (‘case control*’:ti,ab AND random*:ti,ab NOT (‘randomised controlled’:ti,ab OR ‘randomized controlled’:ti,ab)) OR (‘systematic review’:ti NOT (trial:ti OR study:ti)) OR (nonrandom*:ti,ab NOT random*:ti,ab) OR ‘random field*’:ti,ab OR (‘random cluster’:ti,ab AND sampl*:ti,ab) OR (review:ab AND review:it NOT trial:ti) OR (‘we searched’:ab AND (review:ti OR review:it)) OR ‘update review’:ab OR (databases:ab AND searched:ab) OR ((rat:ti OR rats:ti OR mouse:ti OR mice:ti OR swine:ti OR porcine:ti OR murine:ti OR sheep:ti OR lambs:ti OR pigs:ti OR piglets:ti OR rabbit:ti OR rabbits:ti OR cat:ti OR cats:ti OR dog:ti OR dogs:ti OR cattle:ti OR bovine:ti OR monkey:ti OR monkeys:ti OR trout:ti OR marmoset*:ti) AND ‘animal experiment’/exp) OR (‘animal experiment’/exp NOT (‘human experiment’/exp OR ‘human’/exp)))

We utilized Cochrane Highly Sensitive Search Strategy for identifying controlled trials

in Embase: (2018 revision); Ovid format (Glanville et al 2019b)

**Filters:** “randomized controlled trial”; “only Embase results”

Unmarked “Map to preferred term in Emtree”

Unmarked “Search also as free text in all fields”

Unmarked “Explode using narrower Emtree terms”

Unmarked “Search as broadly as possible”

#### Scopus

(TITLE-ABS(“Chorionic Gonadotropin” OR “Recombinant HCG” OR Leuprolide OR “A-43818” OR Enantone OR Leuprolide* OR Leuprorelin OR Lupron OR “TAP-144” OR Buserelin* OR “HOE-766” OR Suprecur OR Suprefact OR Tiloryth OR Nafarelin* OR Synarel OR “Triptorelin Pamoate” OR Triptorelin OR “AY-25650” OR “D-Trp-6-LH-RH” OR Decapeptyl* OR Trelstar OR “Gonadotropin-Releasing Hormone” OR “Luteinizing Hormone” OR “Interstitial Cell-Stimulating Hormone” OR Luteoz* OR Lutropin OR Kisspeptin* OR “KiSS-1 Metastasis Suppressor” OR

“Metastasis Suppressor KiSS-1” OR Metastin*)) AND

(TITLE-ABS(“female infertility” OR anovulat*))

#### Web Of Science

(TS=(“Chorionic Gonadotropin” OR “Recombinant HCG” OR Leuprolide OR “A-43818” OR Enantone OR Leuprolide* OR Leuprorelin OR Lupron OR “TAP-144” OR Buserelin* OR “HOE-766” OR Suprecur OR Suprefact OR Tiloryth OR Nafarelin* OR Synarel OR “Triptorelin Pamoate” OR Triptorelin OR “AY-25650” OR “D-Trp-6-LH-RH” OR Decapeptyl* OR Trelstar OR “Gonadotropin-Releasing Hormone” OR “Luteinizing Hormone” OR “Interstitial Cell-Stimulating Hormone” OR Luteoz* OR Lutropin OR Kisspeptin* OR “KiSS-1 Metastasis Suppressor” OR “Metastasis Suppressor KiSS-1” OR Metastin*))

AND

(TS=(“female infertility” OR anovulat*))

#### LILACS

(mh:(D06.472.699.322.326* OR D06.472.699.327.740.320.400* OR D06.472.699.327.740.320.100* OR D06.472.699.327.740.320.580* OR D06.472.699.327.740.320.790* OR D06.472.699.327.740.320* OR D06.472.699.322.576.463* OR D12.644.276.836*) OR tw:(“Chorionic Gonadotropin” OR “Gonadotropina Corionica*” OR “Recombinant HCG” OR Leuprolid* OR “A-43818” OR Enantone OR Leuprolide* OR Leuprorelin OR Lupron OR “TAP-144” OR Buserelin* OR Busserrelina OR “HOE-766” OR Suprecur OR Suprefact OR Tiloryth OR Nafarelin* OR Synarel OR Triptorelin* OR “Pamoato de Triptor*” OR “AY-25650” OR “D-Trp-6-LH-RH” OR Decapeptyl* OR Trelstar OR “Gonadotropin-Releasing Hormone” OR “Hormônio Liberador de Gonadotropina” OR “Hormona Liberadora de Gonadotropina” OR “Luteinizing Hormone” OR “Hormônio Luteinizante” OR “Hormona Luteinizante” OR “Interstitial Cell-Stimulating Hormone” OR Luteoziman OR Luteozyman OR Lutropin OR Kisspeptin* OR “KiSS-1 Metastasis Suppressor” OR “Metastasis Suppressor KiSS-1” OR Metastin*))

AND

(mh:(C13.351.500.365.700* OR C13.351.500.056.630.050*) OR tw:(“female infertility” OR

“infertilidade feminina” OR “Infertilidad Femenina” OR anovula*))

**Filter:** “Controlled Clinical Trial”

### Appendix

**Supplementary Appendix 2:** Characteristics of excluded studies.

Author, year	Reason for exclusion
Harrison & O’Moore, 1983	Crossover trial
Zreik *et al*., 1999	Crossover trial
Sayyah-Melli *et al*., 2012	Trigger was used in all arms
Masrour & Azad, 2018	Trigger was used in all arms
Thabet *et al*., 2024	Trigger was used in all arms

### Appendix

**Supplementary Appendix 3:** Characteristics of included studies. RCT: randomized controlled trial, hMG: human menopausal gonadotropin; hCG: human chorionic gonadotropin; IUI: intrauterine insemination; CC: clomiphene citrate; US: ultrasound; P: progesterone.

Author, year	Study type	Country	Age	Eligibility criteria	Sample size^1^	Type of infertility	Natural intercourse	IUI	Ovulation induction agent	Intervention group	Control group	Outcomes
Thomas *et al.*, 2019	RCT	India	29.6±3.5 (intervention group) 28.9±3.8 (control group)	Women <36 years, with unexplained infertility, mild endometriosis, mild male factor infertility or polycystic ovarian syndrome, and with at least one patent tube documented following hysterosalpingography or laparoscopy.	392	Unexplained		x	hMG (75-150 IU daily)	hCG (5000 IU)	No intervention	linical pregnancy, ongoing pregnancy, live birth, multiple pregnancy, miscarriage
George *et al*., 2007	RCT	India	24.65±3.48 (intervention group) 25.10±3.99 (control group)	Anovulatory women, diagnosed by cycle length of more than 35 days or a serum progesterone value of less than 10 ng/mL done on the 21st day of the cycle in women with a regular 28-day cycle.	180	Anovulatory	x		CC (100-200mg daily)	hCG (5000 IU)	No intervention	Ovulation (US), ovulation (P), ovulation (US/P), clinical pregnancy, biochemical pregnancy, pregnancy, live birth, miscarriage
Lewis *et al*., 2006	RCT	United States	33.96±3.91 (intervention group) 33.47±3.93 (control group)	Ovulatory women with infertility (defined by at least 1 year of unprotected intercourse without pregnancy, or three failed cycles of donor intrauterine insemination) and with at least one normal, patent fallopian tube and a functional ipsilateral ovary. Women were assumed to have ovulatory cycles if they had monthly menses and biphasic basal body temperature charts, 133or a history of pos363itive ovulation predictor kits, or midluteal serum progesterone levels in the ovulatory range.	150	Unexplained		x	CC (100mg daily)	hCG (10000 IU)	No intervention	Pregnancy rate per cycle, pregancy rate per patient
Yilmaz *et al*., 2006	RCT	Turkey	26.70±3.20 (intervention group) 26.20±3.40 (control group)	Normoprolactinemic and normogonadotropic (WHO Class II ovarian dysfunction) primary infertility with oligomenorrhea (bleeding intervals between 35d and 6mo) or amenorrhea (bleeding interval>6mo), age 20-40, duration of primary infertility≥2years, no history of ovulation induction or thyroid disease, normal results on hysterosalpingogram, and husband with normal semen analysis.	133	Anovulatory	x		CC (50mg daily)	hCG (10000 IU)	No intervention	Clinical pregnancy, ovulation, pregnancy, implantation rate, fertilization rate, twin rate, abortion, midluteal phase level, luteal phase length
Al-Snafi *et al*., 2003	RCT	Iraq	23 to 43	Hiperprolactinemic women with healthy husbands, as shown by semen analysis. Patients with thyroid or adrenal problems, as well as those who used dopamine antagonists, cimetidine and methyldopa, were excluded.	363	Anovulatory	x		Bromocriptine + CC (50mg 3 times a day)	hCG (5000 IU)	No intervention	Pregnancy

1 = only the subset of participants who met our study eligibility criteria are being displayed.

### Appendix

**Supplementary Appendix 5.** Relevant comments from the authors regarding the judgments for each outcome of each study included, using the Cochrane risk-of-bias tool for randomized trials (RoB 2) tool. As the judgments were the same for different results from the same study, the comments are presented only once. However, the table should be read as if only one of the results was being evaluated.

	Randomization	Deviations from intervention	Missing outcome data	Measurement of the outcome	Selection of reported result
Al-Snafi (2003)	No allocation sequence concealment. Because of a preference for ovarian stimulation, especially hormonal stimulation followed by ovulation induction (by patients and gynecologists), the numbers of the second, third and fourth groups were increased compared with the first group.	Participants and personnel were aware of the assigned intervention. We have no information regarding deviations that might have arisen because of the trial context.	---	---	---
Yilmaz (2006)	---	Participants and personnel were aware of the assigned intervention. There were changes from assigned intervention that are inconsistent with the trial protocol, but these are consistent with what could occur outside the trial context.	Sensitivity analyses conducted by review authors show that statistically significant results (*p*≤0.05) are not obtainable under a range of plausible assumptions about the relationship between missingness in the outcome and its true value.	Since it is an outcome that does not involve judgement, assessment of the outcome could not have been influenced by a possible knowledge of intervention received.	---
Lewis (2006)	---	Participants and personnel were aware of the assigned intervention. There were changes from assigned intervention that are inconsistent with the trial protocol, but these are consistent with what could occur outside the trial context.	Sensitivity analyses conducted by review authors show that statistically significant results (*p*≤0.05) are not obtainable under a range of plausible assumptions about the relationship between missingness in the outcome and its true value.	Since it is an outcome that does not involve judgement, assessment of the outcome could not have been influenced by a possible knowledge of intervention received.	---
George (2007)	---	Participants and personnel were aware of the assigned intervention. We have no information regarding deviations that might have arisen because of the trial context.	Sensitivity analyses conducted by review authors show that statistically significant results (*p*≤0.05) are not obtainable under a range of plausible assumptions about the relationship between missingness in the outcome and its true value.	Since it is an outcome that does not involve judgement, assessment of the outcome could not have been influenced by a possible knowledge of intervention received.	---
Thomas (2019)	---	Participants and personnel were aware of the assigned intervention. There were changes from assigned intervention that are inconsistent with the trial protocol, but these are consistent with what could occur outside the trial context.	Sensitivity analyses conducted by review authors show that statistically significant results (*p*≤0.05) are not obtainable under a range of plausible assumptions about the relationship between missingness in the outcome and its true value.	Since it is an outcome that does not involve judgement, assessment of the outcome could not have been influenced by a possible knowledge of intervention received.	---
